# Iron Oxide Nanoparticles Conjugated to Thiosemicarbazone Reduce the Survival of Cancer Cells by Increasing the Gene Expression of *MicroRNA let-7c* in Lung Cancer A549 Cells

**DOI:** 10.34172/aim.2022.126

**Published:** 2022-12-01

**Authors:** Alireza Habibi, Nesa Bakhshi, Zeinab Moradi shoili, Nour Amirmozafari

**Affiliations:** ^1^Departman of Basic Sciences, Faculty of Science, Imam Hossein University, Tehran, Iran; ^2^Departman of Molecular Cell Biology, Faculty of Science, Islamic Azad University of Lahijan, Lahijan, Iran; ^3^Departman of Chemistry, Faculty of Science, University of Guilan, Rasht, Iran; ^4^Departman of Microbiology, School of Medicine, University of Medical Sciences, Tehran, Iran

**Keywords:** Apoptosis, A549 cells, Fe_3_ O_4_@Glu/ BTSC, Lung cancer, MicroRNA let-7c

## Abstract

**Background:**

Cancer cells have a higher demand for iron to grow and proliferate. A new complex of iron nanoparticles and thiosemicarbazones was synthesized. Confirmation tests included UV-visible, scanning electron microscopy (SEM), energy dispersive X-ray analysis (EDX), Fourier transform infrared (FTIR), X-ray diffraction (XRD) and zeta potential.

**Methods:**

MTT assay, flow cytometry and qRT-PCR were used to investigate anti-proliferative effect, amount of apoptosis and the effect of Fe_3_ O_4_@Glu/BTSC on changes in gene expression of *microRNA let-7c* (*let-7c*), respectively. The specifications of Fe_3_ O_4_@Glu/BTSC were confirmed at 5 nm.

**Results:**

Fe_3_ O_4_@Glu/BTSC was more effective than BTSC and Fe_3_ O_4_ on A549 cells (IC_50_=166.77 µg/mL) but its effect on healthy cells was smaller (CC_50_=189.15 µg/mL). The drug selectivity index (SI) was calculated to be 1.13. The initial apoptosis rate was 46.33% for Fe_3_ O_4_@Glu/BTSC, 28.27% for BTSC and 26.02% for Fe_3_ O_4_. BTSC and BTSC@Fe_3_ O_4_ inhibited the cell cycle progression in the Sub-G1 and S phases. *let-7c* expression was 6.9 times higher in treated cells compared to the control group. The expression rate was 2.2 with BTSC compared to the control group and 1.6 times for Fe_3_ O_4_.

**Conclusion:**

Fe_3_ O_4_@Glu/BTSC has proper anti-proliferative effects against lung cancer cells by increasing the expression of *let-7c* and inhibiting the cell cycle with the apoptosis activation pathway.

## Introduction

 There is convincing evidence that mutations in different genes lead to lung cancer (OMIM: 211980). The most important of these genes include *PI3K* (OMIM: 601232), *BRAF* (OMIM: 164757), *EGFR* (OMIM: 131550), *KRAS* (OMIM: 190070), *HER2* (OMIM: 16470), *MEK* (OMIM: 600982) proto-oncogenes. These mutated genes encode proteins that are involved in signaling tyrosine kinase receptors (RTK/OMIM: 602465) and lead to proliferating non-small cell lung cancer.^1^ But the most worrying and common mutations associated with lung adenocarcinoma are mutations in the genes of the EGFR (30%) signaling pathway, such as KRAS.^2^ Other changes effective in the incidence of lung cancer are mutations in the *BAX* (OMIM: 600748) and *BCL2* (OMIM: 603167) genes that lead to cancerous lung tissue in the mitochondrial apoptotic pathway.^3^ Although most researchers have focused on changes in these kinds of genes, one fact in the incidence of cancers such as lung cancer is the interference of some other genes, such as microRNAs. These genes are small non-coding single strands containing 20 to 22 nucleotides and often act as a negative regulator of gene expression after the transcription process. MicroRNA is involved in several cellular processes such as proliferation, metabolism, differentiation, apoptosis or programmed cell death, and epithelial-mesenchymal transmission. Regarding the role of microRNAs as effective factors in regulating gene expression, if the synthesis and function of these molecules are impaired, the homeostasis of organisms can be altered. Several studies have shown that microRNAs get mutated or change their expression in human cancers. These genes were known as the expression regulator of oncogenes and tumor suppressor genes. Some of the most important microRNAs involved in lung cancer are *mir-494* (OMIM: 616036),^4^ *mir-153* (OMIM: 605861),^5^ *mir-101*(OMIM: 605861)^6^ and *let-7c* (OMIM: 612144).^7^ The *let-7* family contains 21 nucleotides whose expression is reduced in the head and neck, prostate, breast, ovary and lung cancers. This decrease in expression leads to a lack of negative regulation in *KRAS*, *C-MYC* (OMIM: 190080), *CDK6* (OMIM: 603368), *HOXA9* (OMIM: 142956), *TGFBR1* (OMIM: 142956), *BCL-XL* (OMIM: 600040), *MAP4K3* (OMIM: 604926) oncogenes. On the other hand, reducing the expression of these genes increases the resistance of cancer cells to some drugs. On the other hand, when the expression of these genes increases, the resistance rate to these drugs decreases. It has been shown inducing the expression of microRNAs *mir-200b* (OMIM: 602091) and *let-7c* significantly reduces the resistance of A549 cells (OMIM: 619408) to erlotinib.^8^

 In recent years, some scientists in the field of genetics and molecular cell biology have focused their attention to other pathways of expression of these genes. One of these pathways is the association between the expression of these genes and the activity of some cell lines, such as *lin-28* (OMIM: 6111043). It has been shown that mutations in the genes of this cell line act as a repressor of *let-7c* biogenesis.^9^ Despite good advances in the study of microRNAs, there is still little data about this issue, including the effect of some compounds on changes in expression of these genes or the role that these genes play in the metabolism of various molecules and ions.

 One of the most important physiological effects of the *let-7c* gene is the regulation of body iron; this gene can apply its effect through binary metals passage channel.^10^ Another effect is regulating and controlling various pathways of apoptosis, for example by inducing it.^11^ On the other hand, some chemical compounds such as thiosemicarbazones can affect the expression of these genes, which is used in the treatment of cancers.^12^ Mainly, thiosemicarbazones metal complexes exert their inhibitory action on various cancers via inhibiting a vital enzyme for DNA biosynthesis and cell division called ribonucleotide diphosphate reductase.^13^ By inhibiting the ribonucleotide reductase enzyme that is an iron-dependent enzyme, compounds containing thiosemicarbazones promote ribose to deoxyribose reduction, block cell cycle synthesis phase and eventually lead to cell death and apoptosis. These compounds are a good option for iron chelation due to their significant lipophilic properties, ability to produce reactive species and inhibition of ribonucleotide reductase enzyme.^14^ Iron is an essential micronutrient for many cellular processes such as oxygen transfer, biosynthesis, DNA synthesis, and cell proliferation, and because of its ability in converting to oxide and reduced forms, it is electron-giving and electron-accepting in nature and is considered a key cofactor for many enzymes. Therefore, depriving cancer cells of iron using iron chelators can be used as a treatment strategy against cancer. Thiosemicarbazonescan chelate iron through their cyclic ligand and deprive cancer cells of this substance by absorbing it and causing cell death. In this study, the interaction of *let-7c* and iron oxide nanoparticles, which was functionalized by a type of thiosemicarbazone, was studied using laboratory methods.

## Materials and Methods

###  Synthesis of Fe_3_ O_4_@Glu/BTSC Nanoparticles

**Figure 1:**
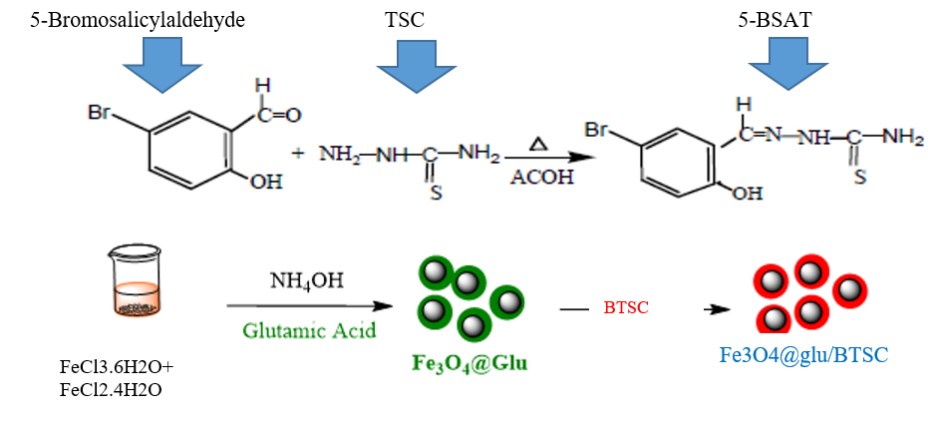
Overview of Synthesis and Functionalization STEPS: A) BTSC, B) Fe_3_ O_4_@Glu/BTSC Nanoparticles.

 Our previous methodology was used to synthesize iron oxide nanoparticles and conjugating them with 5-bromosalicylaldehyde thiosemicarbazone.^15^ The final composition was called Fe_3_ O_4_@glu/BTSC. An overview of the synthesis and functionalization of Fe_3_ O_4_ via BTSC is shown in Figure 1.

###  Identification of Fe_3_ O_4_@Glu/BTSC Nanoparticles

 Fourier transform infrared (FTIR) analysis (Nicolet IR 100 Thermo Scientific Japan) was used to identify the vibrations of functional groups in Fe_3_ O_4_@Glu/BTSC nanoparticles. SEM was used to determine the morphological characteristics and size of the synthesized nanoparticle. Energy dispersive X-ray analysis (EDX) was used to determine the constituent elements of nanoparticles. X-ray diffraction (XRD) analysis )JEOL JDX-803 Japan) was used for fuzzy analysis and examining the size of nanoparticles. After identifying, the crystalline phase of Fe_3_ O_4_@Glu/BTSC nanoparticles was matched to the reported standard patterns. Particle size was also measured using the Scherrer equation. The surface charge of BTSC nanoparticles on iron oxide nanoparticles was evaluated using zeta potential analysis )ZEN3600 MALVERN apparatus, England). In this test, deionized water was used as the dispersant solution. Finally, as a complementary test, the light absorption spectrum of the synthesized nanoparticles (UV-visible) was measured using a spectrophotometer (Inc. UVS-2700) in the range of 1100 to 190 nm. This range is between 250 and 350 nm for iron oxide nanoparticles.^16^

###  Cell Culture

 The lung cancer A549 cell line (http://en.IBC.ir/, accession cell no: IBRC C10080) and human dermal fibroblast cell line (HDF1 BOM, http://ibrc.ir/index.aspx?&siteid=1&pageid=931&p=3&showitem=24) were purchased from the Iranian Biological Resources Center. Cell culture was performed using the method described by DeliveReD.^17^ A549 cells were cultured in the 1640 RPMI medium containing 10% FBS bovine fetal serum and 0.05 mg/mL penicillin G and streptomycin 0.08 mg/mL antibiotics and placed in a wet incubator (55%) overnight at 37°C and 5% CO_2_. To investigate the effect of Fe_3_ O_4_@Glu/BTSC nanoparticles on normal cells, HDF1 BOM that has cross-talk with lung cancer cell line,^18^ was used on Dulbecco’s modified Eagle medium containing 1% penicillin and FBS containing 10% Streptomycin according to the protocol of Kirschner et al and incubated at 37°C for 48 hours.^19^ To prevent cell over-proliferation, 10 μg/mL Mitomycin was added to the medium and placed in an incubator for 3 hours and put in a freezer at 20°C for later use.

###  Evaluation of Cell Survival and Proliferation Using the MTT Method

 According to a study by Zhaoet al, MTT assay was used to evaluate the toxic effects of BTSC, Fe_3_ O_4_nanoparticles and Fe_3_ O_4_@Glu/BTSC complex on A549 cells.^20^ Normal cells of the HDF1 BOM line were used as control cells. *CC50*(cancer cells)/*IC50*(normal cells) was used to calculate the selectivity index (SI) of Fe_3_ O_4_@Glu/BTSC nanoparticles.^21^ In brief, 2 × 10^4^ cells were seeded in each well from a 96-well plate. The cells were then treated with concentrations of 1, 5, 10, 20, 30, 40, 50, 100, 200, 500 μg/mL and placed in an incubator at 37ºC with 5% CO_2_ for 24 hours. 100 µL medium of 0.5 mg/mL MTT solution was added to each well and incubated for 4 hours under the above conditions. Observation of the blue crystals of Formazan dye confirmed that the test was positive. To dissolve the formazan crystals, 200 μL of 1% dimethyl sulfoxide (DMSO) solution was added to each well. After 20 minutes of shaking, the light absorption of the solution obtained at 590 nm was read by ELISA reader (DNM-9602G) and the number of cells was calculated using a standard curve. The IC_50_ value for all three compounds on the A549 cell line and the CC_50_ value for the normal cell line were calculated using GraphPad Prism V5.0 software.

###  Evaluation of Apoptosis and Necrosis by Flow Cytometry Test

 According the protocol of the manufacturer company (Apoptosis detection kit, Roche, Switzerland, Germany), Fluorescein-Annexin V isocyanate and propidium iodide were used to evaluate the effect of synthesized compounds on different phases of the cell cycle, apoptosis and necrosis. A total of 10^6^ cells were seed in six-well plates and treated with an IC_50 _concentration of the synthesized material. They were then placed in an incubator at 37°C and 5% carbon dioxide for 24 hours. After trypsinization, the cells were washed with phosphate-buffered saline (PBS, Sigma-Aldrich). 10 µL of propidium iodide dye and 5 µL of Annexin-V dye were added to the content of the micro-tube. After 10 minutes of incubation at room temperature, cell analysis was performed by flow cytometry device and compared with the control group (untreated cells).^22^

###  Quantitative Reverse-Transcriptase PCR Assay (QRT-PCR)

 In the present study, based on the study by Pearce et al, the *let-7c* gene expression level was measured using qRT-PCR analysis SYBR Green.^23^ TransGen Biotech TransZol Kit (Beijing TransGen Biotech Co., Ltd., China) manufacturer instruction was used to extract the total RNA. Briefly, after culturing A549 cells in a 12-well plate, 1 mL of TransZol was added to each well and after 5 minutes of incubation at room temperature, the cells were transferred to an RNase-free microcentrifuge. 0.1 mL of chloroform was added to each micro-fusion and placed in a shaker for 15 seconds. The incubation was then performed for three minutes at 4°C and the content of the tube was centrifuged at 10 000 rpm/min for 10 minutes. Then, 0.25 mL of isopropyl alcohol was added to the supernatant. The supernatant was separated and then 1 mL of 96% alcohol was added to the tube. Centrifuge was done at 8000 rpm/min at 4°C for 15 minutes and the supernatant was discarded. To dissolve sediments of the bottom of the tube, 1-2 mL of RNA lysing solution was added and incubated at room temperature for one minute. After centrifugation at 12 000 rpm/min for one minute, the supernatant was divided into small volumes and stored at -80°C. The concentration of extracted RNA was evaluated using a US-made nano-drop spectrophotometer, at the wavelength A260/A230.

 The protocol of the manufacturer company of EasyScript First-Strand cDNA Synthesis SuperMix kit (TransGen Biotech, Beijing, China) was used for cDNA synthesis. To start synthesis, 5 μg of total RNA, 1 μL of Anchored oligo (DT), 18 primers (0.5 µg/µL) and 20 µL of deionized water were mixed. Then, incubation was performed for five minutes at 65°C. The micro-tubes were then placed on ice for two minutes. Then, other compounds including 1 μL EasyScript RT/RI Enzyme Mix and 10 μL 2 × ES Reaction Mix were added to the microtube. The final mixture was incubated at 42°C for 15 minutes and maintained at -80°C.

 To perform the qRT-PCR reaction, a pair of *let-7c* primers were used with the following sequence: F: 5´-GGTTGAGGTAGTAGGTTGTATGGT-3´ and 5´-AACATGTACAGTCCATGGATG-3´.^24^ QRT-PCR reactions were performed after evaluation and measurement of their length by electrophoresis on 2.5% agarose gel, using SYBR Premix Ex Taq TM Kit (TaKaRa, Dalian, China) and (ABI 7500 real-time PCR system Applied Biosystems, USA) apparatus. The *GAPDH* (OMIM: 134800) host gene was used with sequence F: 5′-CCCACTCCTCCACCTTTGAC-3′ and R: 5′-CATACCAGGAAATGAGCTTGACAA-3′ as a control gene.^25^ The temperature cycle conditions based on the protocol of the manufacturer company of the above kit are summarized in Table 1. All reactions were repeated three times and the expression status of the target genes was compared with the mRNA expression level of the *GAPDH* gene using the 2^−ΔΔC^_T_ method.

**Table 1:** qRT-PCR Program and Reaction Steps

**Reaction Stage**	**Time**	**Temperature (°C)**	**Cycle**
Initial denaturation	5 s	95	1 cycle
Denaturation	15 s	95	30 cycles
Annealing	5 s	XX	
Extension	1 min	72	
Final extension	5 min	72	1 cycle

###  Investigation of the Effect of Synthesized Materials on Cell Cycle Phases

 The effect of Fe_3_ O_4_@Glu/BTSC on G0/G1, S and G2/M phases was evaluated according to the method described by Davy and Doorbar.^26^ After culture, the cells were treated with concentrations of 10–20 μL of Fe_3_ O_4_@Glu/BTSC and 0.05% DMSO (as negative control) for 15 hours. Rinsing was performed with PBS and kept in a -20°C freezer for 24 hours with the addition of 70% alcohol overnight. To perform flow cytometry, the cells were washed again with cold PBS and suspended for 15 minutes at room temperature by adding 50 µL of RNase A 100 μg/mL. In the next step, propidium iodide was added to the suspension to reach the final concentration of 20 μg/mL. The suspension was placed at room temperature for 20 minutes. Cell cycle phases were analyzed by FACS Scan Flow Cytometer (Becton-Dickinson CA, USA). Data were analyzed with three replications, and different phases of the cell cycle were analyzed using flowing software 2.5.1.

###  Data Analysis

 Data analysis was performed in GraphPad Prism 5 software with ANOVAtest and a significant value was accepted and analyzed at error level *P* < 0.05.

## Results

###  Analysis of Synthesized Complex

 The FTIR test results for Fe_3_ O_4_@Glu/BTSC are as follows. Peaks at 3400 and 3500 indicated the symmetrical vibration of N-H related to the NH_2_ group on the BTSC.^27^ The peak at 1990–2140 indicated the presence of the thioamide group (N-C = S) unconnected to the aromatic compound.^28^ The 3500 range is related to the N-H band,^29^ which was not present in the material under study and this point indicated the binding of Fe_3_ O_4_ nanoparticles to BTSC. The peak of 1690 is related to the C = N band. The range 1650-2000 is related to the C-H bands located in the aromatic ring. These groups are of the tensile type. The C = C peak bands of cyclic compounds are in the range 1566–1650. In synthesized thiosemicarbazone, the C = C band was located in the aromatic ring, which corresponded to the 1628 peak. This peak is also of the stretching type.^30^

**Figure 2:**
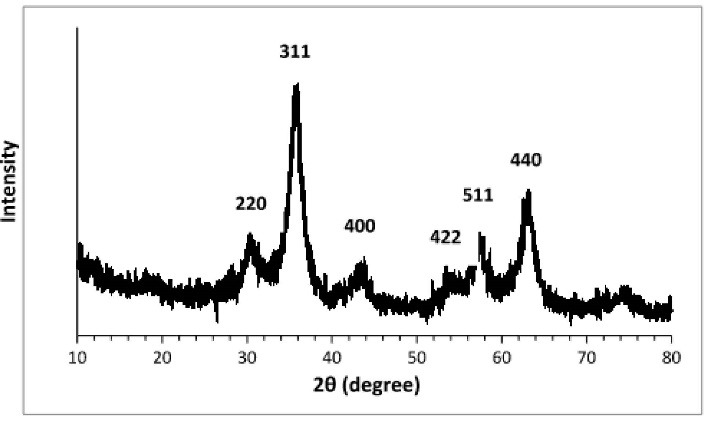
X-Ray Diffraction Pattern of Fe_3_ O_4_@Glu /BTSC Nanoparticles

 The XRD spectrum of Fe_3_ O_4_@Glu/BTSC nanoparticles is shown in Figure 2. Common reflective plates including (220), (311), (400), (422), (511), (440) at 2θs of 30/11˚, 36/05˚, 43/98˚, 53/98˚, 57/98˚ and 63/85˚, respectively were observed that indicate the cubic phase of the full faces centers. The diffraction patterns of the specific peaks matched with the 750033-JCPDS code. The Scherrer equation was used to calculate the particle size which was obtained at about 5 nm.

**Figure 3:**
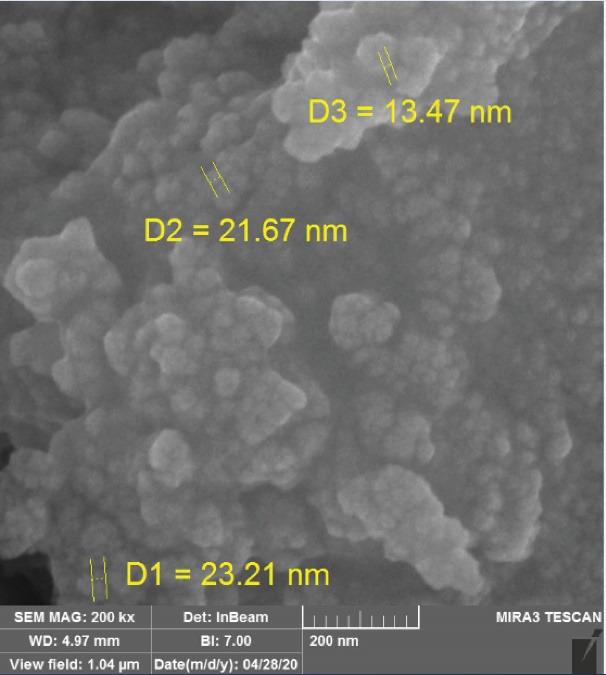
SEM Image Related to Fe_3_ O_4_@Glu/BTSC Nanoparticles

 The morphology of the nanoparticles was determined using a scanning electron microscope (SEM). SEM images related to Fe_3_ O_4_@Glu/BTSC synthesized nanoparticles showed that these nanoparticles were spherical (Figure 3). According to the results, the size of the synthesized nanoparticles was uniform and was about 13-23 nm on average. The cause of the difference in size calculated in the XRD method and the Scherrer equation and the sizes observed in the SEM images can be attributed to the relative aggregation of the synthesized nanoparticles.

**Figure 4:**
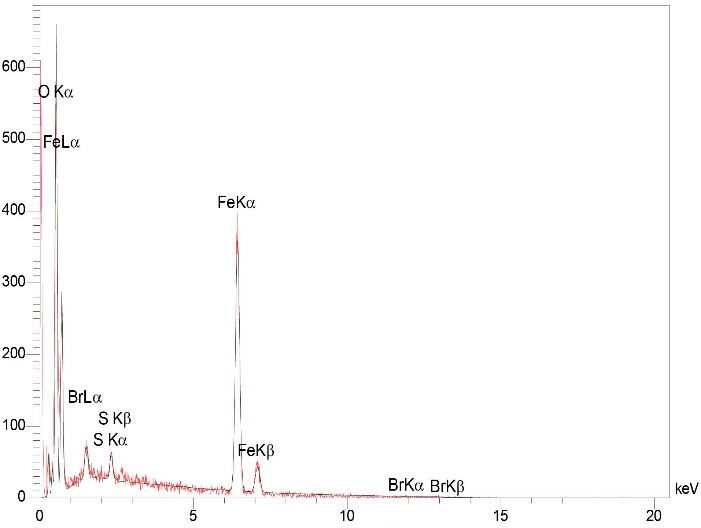
EDX Analysis of Fe_3_ O_4_@Glu/BTSC Nanoparticles

 The results of EDX analysis confirmed the presence of Br, Fe, S, C, N and O in Fe_3_ O_4_@Glu /BTSC. The presence of these elements and the absence of peaks of other elements in the EDX spectrum confirmed the purity of the synthesized complex (Figure 4).

**Figure 5:**
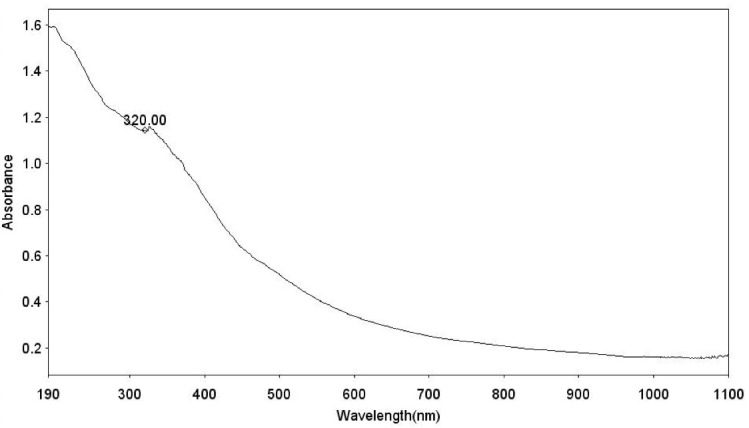
UV-Visible Analysis of Fe_3_ O_4_@Glu/BTSC Nanoparticles

 The UV-visible analysis of the synthesized Fe_3_ O_4_@Glu/BTSC nanoparticles is shown in Figure 5. The optical absorption spectrum of iron oxide nanoparticles was observed at 320 nm and this peak indicates the formation of iron oxide nanoparticles and is consistent with the results of Ullah et al.^31^

**Figure 6:**
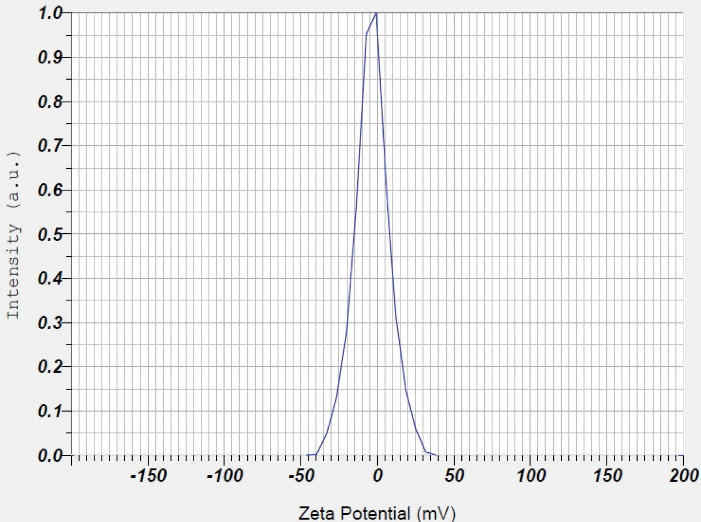
Results of Zeta Potential of Fe_3_ O_4_@Glu /BTSC Nanoparticles

 The results of zeta potential analysis in deionized water for Fe_3_ O_4_ nanoparticles with BTSC coating were equal to -3.5 mV (Figure 6). In fact, the thiosemicarbazone coating stabilizes iron oxide nanoparticles. The value of zeta potential in colloidal solutions with suitable stability is equal to -30- + 30 mV. This rate of zeta potential can provide electrostatic repulsion force between nanoparticles and leads to their stability.^32^ Therefore, the rate of zeta potential in our test was appropriate.

### Fe_3_ O_4_@Glu/BTSC Inhibits Lung Cancer A549 Cell Growth in a Concentration-Dependent Process

**Figure 7:**
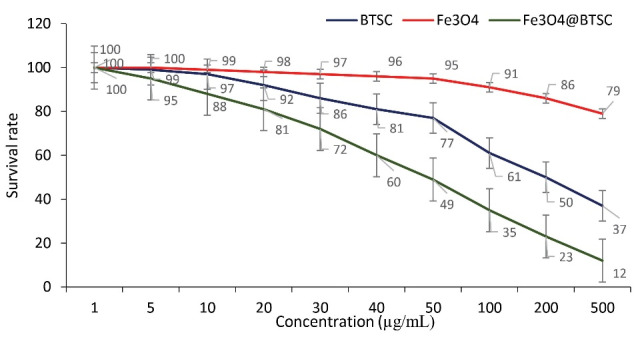
Effect of BTSC, Fe_3_ O_4_ and Fe_3_ O_4_@Glu /BTSC on A549 Cells

MTT test was used to evaluate the effect of synthesized compounds on lung cancer cell line (A549) and normal cell line (HDF1 BOM). Using the results of this test, IC_50_ and CC_50_ values were calculated. The results of this test confirmed the anti-proliferative activity of BTSC, nanoparticle and Fe_3_ O_4_@Glu/BTSC on the growth of cancer cells. But the effect of these compounds was different on the two cell groups; for cancer cells, the IC_50_ values for BTSC, Fe_3_ O_4_ and Fe_3_ O_4_@Glu/BTSC were 318.51, 1133.34 and 166.77, respectively. These data showed that Fe_3_ O_4_@Glu/BTSC was effective on A549 cells at a much lower dose than the other two substances, and this was also the same for BTSC compared to Fe_3_ O_4_ (*P* < 0.05; Figure 7).

In the case of normal HDF1 BOM cells that were treated with these three compounds for 48 hours, the results were similar to those seen in A549 cells. But the CC50 of these compounds on these cells was higher than that of A549 cells. The CC50 values ​​for the three compounds BTSC, Fe_3_ O_4_ and Fe_3_ O_4_@Glu /BTSC were 356.25, 1210.62 and 189.15, respectively.

**Figure 8:**
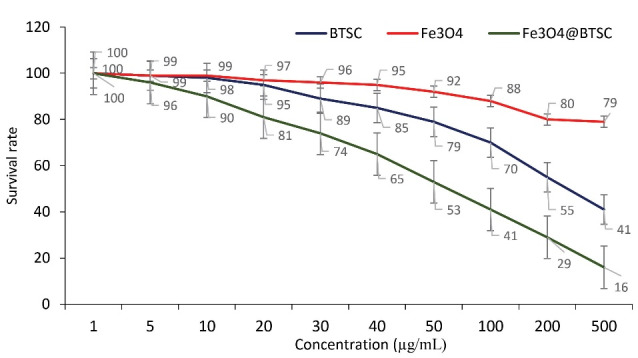
Effect of BTSC, Fe_3_ O_4_ and Fe_3_ O_4_@Glu /BTSC on HDF1 cells

The results showed that at a concentration of CC50 = 18915 μg/mL, this drug had a cytotoxic effect on cells (Figure 8). Therefore, at lower concentrations of the drug, the rate of cell survival was higher. The SI obtained was equal to 1.13.

### Fe_3_ O_4_@Glu/BTSC Induces Apoptosis in a Higher Proportion Than BTSC and Fe_3_ O_4_

**Figure 9:**
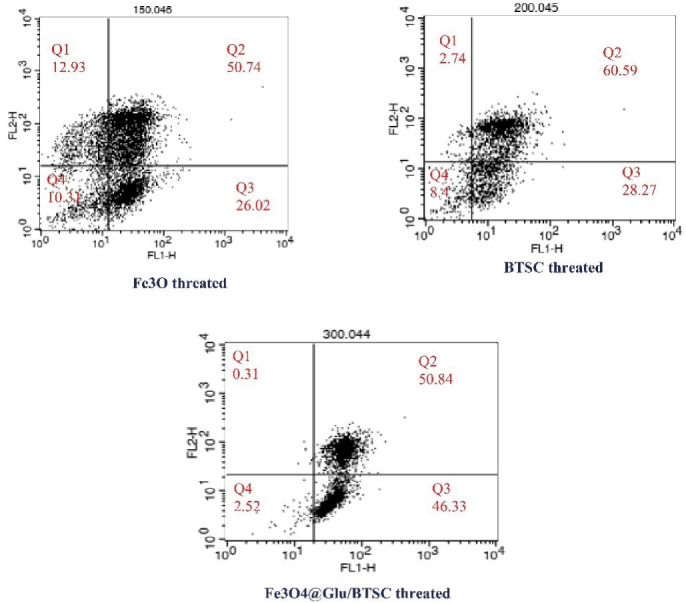
Results of Annexin V Staining Assay Protocol in A549 Cancer Cells

 Annexin V/PI staining and flow cytometry were used to evaluate the effect of Fe_3_ O_4_@Glu/BTSC on apoptosis. In this test, the percentage of living cells before treatment with Fe_3_ O_4_@Glu/BTSC was 95% and the percentage of living cells after treatment with different compounds is shown in Figure 9. In the present study, after treating the cells with Fe_3_ O_4_, the initial apoptosis (Q3) was 26.02%; it was 28.27% with BTSC and 46.33% with Fe_3_ O_4_@Glu/BTSC. Regarding early and late apoptosis (Q2 + Q3), Fe_3_ O_4_@Glu /BTSC was able to induce 97.17% apoptosis in A549 lung cancer cells. This ratio was lower for the other two substances (*P* < 0.05). The necrosis value for Fe_3_ O_4_@Glu/BTSC was 0.31% (Q1). Also, this value was only 2.52% for living cells after treatment with the recent substance, which was much lower than the other two substances (Q4).

###  Fe_3_ O_4_@Glu/BTSC Nanoparticles Increase let-7c Gene Expression

**Figure 10:**
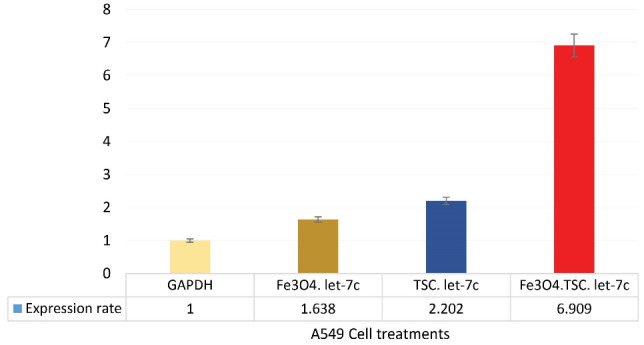
Changes in the Expression of *let-7c* Gene in Treated Cells and Control Gene

 The results of the qRT-PCR test showed that Fe_3_ O_4_ and BTSC compounds increase *let-7c* expression. *Let-7c* gene expression in nanoparticle-treated cells increased 1.6-times in 24 hours compared with the *GAPDH* control group. When the cells were treated with BTSC, the expression level of this gene reached 2.2 compared to the control group. However, after iron nanoparticles were conjugated with BTSC, the expression level increased to 6.9- times (Figure 10).

###  Fe_3_ O_4_@Glu/BTSC Nanoparticles Increase Cell Population in S and Sub-G1 Phases

 The results of the effect of the synthesized materials were an increase in cell population in the S phase of treated cells with nanoparticles and a decrease in cell population in treated cells with Fe_3_ O_4_ nanoparticles. Flow cytometry was used to determine the relationship between the inhibitory effect of BTSC and Fe_3_ O_4_@Glu/BTSC at different phases of the cell cycle in a concentration-dependent manner. In BTSC-treated cells at two concentrations of 10 and 20 μg, an increase in cell population was observed in Sub-G1 and G1 compared to untreated cells (control group). In Fe_3_ O_4_@Glu/BTSC-treated cells, the cell population showed a significant increase in S and Sub-G1 phases (*P* < 0.05); however, in these cells, compared to untreated cells, G1 showed a decrease in number (Table 2). The results were different about the effect of the synthesized compounds in the S phase; thus, the cell population decreased after treatment with BTSC compared to the control group, but when these cells were treated with Fe_3_ O_4_@Glu/BTSC, their number increased compared to both groups.

**Table 2:** Effect of BTSC and Fe_3_ O_4_@Glu/BTSC on A549 Cell Population in Different Phases of Cell Cycle.

**Cell group**	**Cell Population in Sub-G1 Phase**	**Cell Population in G1 Phase**	**Cell Population in S Phase**	**Cell Population in G2 Phase**
Control	3.2	62.3	28.9	9.8
Treated with BTSC	5.8	65.5	20.7	9.01
Treated with Fe_3_ O_4_@Glu/BTSC	8.3	55.4	31.6	9.03

## Discussion

 Despite the discovery of new methods for cancer treatment, there are still many challenges such as resistance, malignancy, recurrence and threatening negative effects in the treatment of this disease. In the last two decades, scientists have made many efforts against cancer successfully. In many cases, cancer cells counteract with the therapeutic solutions and sometimes, even with the incidence of resistance, cause tumor recurrence. Thiosemicarbazones have been widely used against resistant and non-resistant forms of cancer.^33^ Thiosemicarbazones, which can chelate iron ions, produce active radicals by effectively absorbing iron and affecting various pathways in cancer cells. Numerous studies have been performed on the effect of thiosemicarbazone metal complexes on lung cancer cells. In one study, the success of inhibiting the migration and metastasis of lung cancer cells was reported by a series of (1-4) complexes of thiosemicarbazone nickel nanoparticles.^34^ In another study, conjugation of magnetic nanoparticles with silica layers could promote inhibition of lung cancer cells.^35^ In the present study, the effect of a complex of Fe_3_ O_4_ and 5-bromosalicylaldehyde thiosemicarbazone (Fe_3_ O_4_@Glu/BTSC) nanoparticles on the A549 cell line of lung cancer was investigated. An important goal of this study was to investigate the anti-proliferative activity of this compound on the studied cell line. Another aim of this study was to investigate the changes in the expression of the *let-7c* gene in Fe_3_ O_4_@Glu/BTSC-treated cells. The results of this study showed that Fe_3_ O_4_@Glu/BTSC has proper anti-proliferative activity (166.776 g/mL, IC_50_) against lung cancer A549 cells. Also, by inducing apoptosis, this drug combination, at a concentration of 50 g/mL, was able to inhibit the growth of lung cancer cells compared to untreated cells for 24 hours. The survival percentage at this concentration was 49%. But at a higher concentration (189.15 µg/mL) than CC50, this substance had a cytotoxic effect on HDF1 BOM cell line. The survival percentage at the 100 µg/mL concentration was 41%. According to the value of the SI (1.13), the toxic concentration of the drug in cancer cells was higher than in normal cells.^21^ A comparative study of this compound showed that the SI value for this complex was lower than other conjugated metal derivatives studied in our previous research (3.48). In another study conducted by Marković et al, thiosemicarbazone derivatives were studied on the A549 cancer cell line and these compounds showed a good toxicity effect.^36^ Also, in a study conducted by Sever et al on thiosemicarbazone derivatives against the A549 cell line, the result showed that substitutions on the benzene ring had a significant effect on the cytotoxicity of the compounds.^37^

 Since there are benzene rings with different substitutions in the thiosemicarbazone structure, the Fe_3_ O_4_@Glu/BTSC complex can have a suitable cytotoxicity effect against cancer cells. Another finding of this study was that 5-bromosalicylaldehyde thiosemicarbazone alone had little effect in inhibiting the activity of cancer cells, the main reason being the inability of this substance to penetrate the cells. Iron oxide nanoparticles also showed little anti-proliferative activity against cancer cells. Since the Fe_3_ O_4_@Glu/BTSC complex is an effective compound against lung cancer cells, the findings of this study confirmed the delivery properties of iron oxide nanoparticles and the anti-proliferative properties of thiosemicarbazones. The most obvious finding of the present study was the activation of apoptotic pathways by Fe_3_ O_4_@Glu/BTSC which can probably be attributed to the chelation properties of iron ions by thiosemicarbazone. Experiments based on molecular pathways were performed to prove this hypothesis. Apoptotic cells can be examined using surface markers such as phosphatidylserine. Phosphatidylserine is naturally present on the inner surface of the plasma membrane and is transmitted to the outer surface of the membrane in the early stages of apoptosis and this transmission acts as a signal for the attack of phagocytic cells. The most effective confirmatory test for apoptosis study is the Annexin V Staining Assays. This reagent has a high affinity for binding to phosphatidylserine and its displacement across cell membranes indicates the occurrence of apoptosis. In this study, the results of Annexin V ^+^ /PI - staining in A549 cell line treated with Fe_3_ O_4_@Glu/BTSC showed an increase in early apoptosis ( > 46.33%) compared to cells treated with the other two substances. Since the magnetic iron oxide nanoparticles functionalized with thiosemicarbazone have unique chelation properties, the Fe_3_ O_4_@Glu/BTSC complex is proposed as an agent with anti-proliferative potential against A549 cells of lung cancer. These findings also express that iron oxide nanoparticles, in addition to helping early apoptosis, play an important role in the conduction of anti-cancer ligands of lung cancer. These results are consistent with Du’s findings.^38^ Thus, data related to flow cytometry results suggest that Fe_3_ O_4_@Glu/BTSC activates apoptosis in lung cancer cells. In a study by Subasi et al, the results showed thiosemicarbazones derivatives leading to G0/G1 and G2/M arrest phases in the cell cycle of the colorectal cell lines.^39^ The binding of iron oxide nanoparticles to thiosemicarbazone causes targeted transfer and high solubility of anticancer compounds in the target tissue. These findings are also consistent with the results of the research by Izadpanah et al.^13^ The nano-complex used in this study was able to increase the expression of the *let-7c* gene, which is a tumor suppressor gene. Thus Fe_3_ O_4_@Glu/BTSC can be considered as an inhibitor of the cancer cells growth. In fact, this compound is effective in regulating the high expression of the *let-7c* gene in lung cancer. On the other hand, the high cell population in the Sub-G1 and G1 phases indicates the arrest of the cell cycle by BTSC. The results of cell cycle analysis showed that both BTSC and BTSC@Glu/Fe_3_ O_4_ led to an increase in the inhibition of cell cycle progression in the Sub-G1 phase. This result was consistent with the findings of Izadpanah et al, who studied thiosemicarbazone derivatives and Fe_3_ O_4_ in the breast cancer cell line.^13^ Cell cycle arrest in the Sub-G1 and S phases by BTSC@Glu/Fe_3_ O_4_ describes DNA fragmentation and disintegration, which is associated with further permeability of cells through Fe_3_ O_4_ nanoparticles. Various studies have reported the role of drug delivery by iron nanoparticles.^40^

 Radha and Raghavan showed that DNA fragmentation leads to apoptosis in cells.^41^ Therefore, BTSC@Glu/Fe_3_ O_4_ is suggested as an effective drug in cell apoptosis in Sub-G1 and S phases on the lung cancer cell line.

 In conclusion, the main purpose of this study was to investigate the inhibitory role of Fe_3_ O_4_@Glu /BTSC in different phases of the cell cycle of A549 lung cancer cells and its effect on *let-7c* gene expression and induction of apoptosis. According to the results of this study, Fe_3_ O_4_@Glu/BTSC is a substance with anti-proliferative activity against cancer cells. The synergistic activity of BTSC and Fe_3_ O_4_ nanoparticles also induces expression increased of the *let-7c* gene. Failure to investigate the effect of the synthesized complex on other cancer cell lines and other genes as well as *in vivo* conditions were among the limitations of the present study which are suggested for future research.
